# Mourning ritual participation, subjective well-being and prosocial behaviour among the Luhya people of Kenya

**DOI:** 10.1098/rspb.2025.0213

**Published:** 2025-09-03

**Authors:** Stephen Asatsa, Erik Ringen, Rohan Kapitany, Stephen Mbugua Ngaari, John Makunda, Elizabeth Wangari Gichimu, Sheina Lew-Levy

**Affiliations:** ^1^ Department of Psychology, The Catholic University of Eastern Africa, Nairobi 00200, Kenya; ^2^ Institute for the Interdisciplinary Study of Language Evolution (ISLE), University of Zurich, Zürich CH-8050, Switzerland; ^3^ Department of Psychology, Durham University, Durham DH1 3LE, UK; ^4^ Department of Psychology, Daystar University, Nairobi 00100, Kenya

**Keywords:** mourning rituals, subjective well-being, prosociality, cooperation

## Abstract

This study explores the relationship between participation in mourning rituals, well-being and prosociality within the Luhya community of western Kenya. Cooperation, essential for human success, is often reinforced through rituals that strengthen social bonds and collective identity. We hypothesized that mourning rituals enhance prosocial behaviour, and that this relationship is mediated by subjective well-being. To test these hypotheses, study 1 utilized quantitative surveys to assess the pathway between ritual participation, well-being and prosociality. In support of our hypotheses, we found a strong total effect of ritual participation on prosociality, and strong evidence for the pathway between ritual participation, well-being and prosociality. To contextualize these findings, study 2 employed qualitative methods, including focus groups and interviews, to capture participants’ experiences. Narratives illustrated how mourning rituals facilitated communication, emotional expression and community cohesion. Participants noted that rituals helped process grief and reinforced social ties. However, some rituals were perceived negatively, highlighting their complex emotional impact. Overall, our findings suggest that Luhya mourning rituals are vital for enhancing well-being and fostering prosociality, emphasizing the importance of cultural practices in promoting cooperation across communities.

## 1. Introduction

Cooperation is a fundamental aspect of human success [1]. It is defined as the process through which individuals work together towards a common goal, often requiring mutual support and coordination. While cooperation is observed across animal taxa, from bees to chimpanzees [2], the scope and scale of human cooperation is unique to our species, involving kin and strangers, households and large cities [3]. Our propensity for cooperation is thought to have evolved in response to our complex foraging niche, and to the demands of caring for multiple overlapping dependent offspring [4,5]. Cooperation is thus crucial for the survival of our species, and likely underpins our capacity for cumulative culture, a hallmark of human development upon which knowledge and skill are progressively built [6].

Rituals can be understood as structured, repeated and often non-instrumental actions imbued with cultural significance, serving to reinforce social bonds and shared identities within a group [7–9]. The earliest evidence for rituals in our species is thought to be the collection of ochre, occurring some 300 000 years ago [10]. Rituals may have evolved to improve group cohesion during times of resource and social stress [10]. Such adaptive benefits are evidenced in studies from contemporary societies, where rituals have been shown to enhance cooperative behaviour [11–13]. For example, participation in rituals fostered trust and cooperation among Tyvans in the Tyva Republic [14]. In Hindu-dominated Mauritius, extreme rituals promoted prosociality in participants and observers [15]. In an experimental study, participating in ritualized synchronous behaviour (marching) led groups of men to underestimate a foe’s formidability, and overestimate their own [16]. Three features of ritual may promote cooperation. First, high emotional arousal, especially dysphoria, can strengthen connections among participants, creating shared memories that enhance group cohesion [17–20]. Second, rituals provide reliable markers of group membership and demonstrate commitment to the collective, thus enhancing group cohesion and facilitating social trust [21–23]. Finally, the synchrony often experienced during ritual, such as music and dance, not only promotes social bonding but also enhances cooperative tendencies through shared intentionality [24–26].

The psychological mechanisms underlying the association between ritual and in-group cooperation, however, remain poorly understood [27]. One way in which ritual may promote cooperation is by improving well-being. Various studies have demonstrated that participating in communal rituals improved feelings of belonging, mutual support and perceived health [28–30]. Other studies have shown that well-being and prosociality are linked [31–34], with greater life satisfaction and positive affect consistently predicting prosocial actions across diverse cultures. Finally, communal rituals may foster both psychological benefits and social connections. In Mikasa, Japan, active participation in festival dances had a positive relationship with social cohesion, and observing the festival had a negative association with anxiety [35]. In London, secular Sunday Assemblies and Christian congregations both increased positive affect and promoted feelings of social bonding [36]. A longitudinal study in Prayagraj and Delhi, India, found social bonding patterns were similar in the two settings, indicating that Diwali fosters group cohesion across diverse social ecologies [28].

Considering this research, in the present paper, we posit that the effect of ritual on prosociality is mediated by well-being. We tested this association in two studies conducted among the Luhya community of western Kenya. Our hypotheses and approach were registered on Open Science Framework (https://osf.io/wsycu), with deviations reported in the electronic supplementary material. In both studies, we focus specifically on Luhya mourning rituals. Death represents a profound disruption in societal cohesiveness, often leading to feelings of isolation, fear and uncertainty among community members [37,38]. This rupture creates a vacuum where social support systems weaken, resulting in increased anxiety and diminished collective identity. Mourning rituals serve as essential practices that help re-establish social bonds. These rituals provide a structured framework for expressing grief and commemorating the deceased, allowing individuals to process their loss collectively [39]. Previous studies suggest that engaging in communal mourning fosters in-group solidarity, as shared experiences of grief can strengthen interpersonal ties and enhance social cohesion [40,41]. By participating in these culturally patterned practices, individuals may find solace, regain a sense of belonging and restore their emotional well-being [38]. Cross-culturally persistent mourning rituals include burial, community meals, preaching and memorial services [42–44]. Further, across cultures, specific rituals are performed based on the deceased’s status, gender and manner of death [45–47]. The practice of these widespread mourning rituals in the Luhya community indicates that insights gained from the present study regarding the interplay of ritual, well-being and prosociality are likely applicable to broader contexts.

Study 1 quantitatively tested two hypotheses:

**H1**: if participation in mourning rituals boosts cooperation, then mean prosociality scores will be higher for individuals who have participated in a traditional Luhya mourning ritual.

**H2**: if the effect of ritual on prosociality is mediated by well-being, then participation in traditional Luhya mourning rituals will lead to higher well-being scores which, in turn, will lead to a higher prosociality score.

In study 2, we further unpacked the association between ritual, well-being and cooperation using rich ethnographic data from in-depth interviews and focus group discussions with bereaved adolescents, bereaved adults, community elders and religious leaders selected from three counties inhabited by the Luhya community.

## 2. Ethnographic setting

This study was conducted among the Luhya, the second largest ethnic community in Kenya, making up about 14% of Kenya’s total population [48]. The Luhya rely on agriculture as their main economic activity, though many are professionals in various sectors of the economy. This study targeted the five most populous clans: Bashitsyula, Bukusu, Tiriki, Banyala and Batsotso. They mainly live in the western Kenya counties of Kakamega, Vihiga, Bungoma, Busia and Trans Nzoia. This is a highly patriarchal community, and the status of men in society is still viewed as more esteemed compared with that of women. Given the sensitive nature of the present study, it was essential to involve individuals with an intrinsic understanding of Luhya culture. Accordingly, most team members were from the Luhya community.

The Luhya highly value rituals in their day-to-day life, with major life milestones marked with specific accompanying rituals. Child naming is the responsibility of the paternal grandmother; names are derived from relatives who are highly esteemed in the community, or the season when the child is born. Several days after birth, the first hair of the child is shaved by the grandmother as a way of incorporating the child in Luhya identity [49]. Circumcision is a significant rite marking the transition from boyhood to manhood, often celebrated with communal gatherings and teachings about adulthood [50]. During marriage celebrations, traditional songs and dances are performed, highlighting cultural heritage and fostering communal bonds [51]. Most importantly for the present study, mourning rituals serve as essential expressions of grief and communal solidarity. Upon the death of a community member, families typically observe a mourning period, during which relatives and friends come together to provide support and condolences [51,52]. The burial ceremony is a significant event, often marked by traditional practices such as the washing of the body and the use of specific burial attire, which reflect the deceased’s social status [53]. Following the burial and throughout the year-long mourning period, the community engages in rituals that include communal gatherings and feasting, allowing members to celebrate the life of the deceased and reinforce social ties. The death anniversary (Makumbusho), and destroying the house of the deceased, occurs 1 year after the loss. These mourning practices not only facilitate healing for the bereaved, but also strengthen the community’s collective identity and cultural continuity.

## 3. Study 1: does ritual predict prosociality?

### (a) Methods

#### (i) Procedure and sample

Surveys were used to investigate the pathway between ritual participation, subjective well-being and prosociality. Specifically, respondents were recruited from Kakamega, Vihiga and Bungoma counties. Chiefs, who are well acquainted with local families due to their involvement in issuing burial permits, referred us to households where a bereavement had occurred within the past 12 months. Some families further directed us to other bereaved households. Additionally, adolescents who had experienced the loss of a family member within the past year were identified through local schools. Families were randomly selected from those identified through these channels, and only those who were willing to participate were recruited.

Prior to data collection, we simulated 100 datasets for varying sample sizes and assumed a conservative correlation between participation in a mourning ritual and well-being (0.2), well-being and prosociality (0.2), and ritual participation and prosociality (0.1). With a sample of 250 participants, we were able to detect the pathway between ritual participation, well-being and prosociality with 95% credible intervals (CIs) for over 90% of datasets. Guided by this simulation, we surveyed a total of 261 respondents 15 years and over (58.6% women) who had experienced the loss of a loved one. As most mourning rituals occur in the first year of bereavement, we only recruited individuals who had experienced a loss within the last 12 months. Respondents reported demographic information, answered a questionnaire regarding participation in Luhya mourning rituals and took two psychometric tests (electronic supplementary material, table S1). Surveys were translated and back-translated into Kiswahili, Kenya’s *lingua franca*. The surveys were then piloted with 20 community members and revised to improve clarity based on their feedback. To maximize comprehension, surveys were administered in both Kiswahili and English—Kenya’s language of school instruction—such that all respondents simultaneously saw questions in both languages. Most surveys were administered with pen and paper. If a respondent indicated that they could not read and/or write, a research assistant asked survey questions verbally and recorded responses.

#### (ii) Demographic information

We collected data on demographic variables that we felt could potentially confound associations between ritual participation, subjective well-being and/or prosociality. (i) Age: several studies demonstrate that subjective well-being and prosocial behaviour vary with age [54,55]. In addition, our ethnographic observations suggest that older individuals have greater participation in traditional Luhya mourning rituals. (ii) Sex: research also suggests that subjective well-being and prosocial behaviour vary with sex/gender [56–58]. Further, men play a more central role in Luhya mourning rituals. (iii) Education: we previously found that educational attainment was negatively associated with participation in traditional Luhya rituals because formal educational institutions tend to discredit Indigenous rituals in favour of Christian/Muslim ones [52]. Higher levels of education have also been associated with gains in subjective well-being [59]. (iv) Occupation type: respondents reported their occupation. We then categorized these according to approximate income level. High-income occupations included professional roles such as police officer or teacher. Middle-income occupations included large-scale agriculture. Low-income occupations included subsistence agriculture, informal work and students. Socioeconomic status has previously been associated with subjective well-being and prosocial behaviour [60,61]. (v) Clan territory*:* among the Luhya, clans are associated with specific geographic territories towards which clan members have rights and responsibilities, even when living far from home. These clans are inherited patrilineally. Each clan has its own dialect, and local variations of traditional rituals. Clans vary in size, with some clans more traditional and others more oriented towards Christian/Muslim religious practices.

#### (iii) Ritual participation

**Figure 1.**
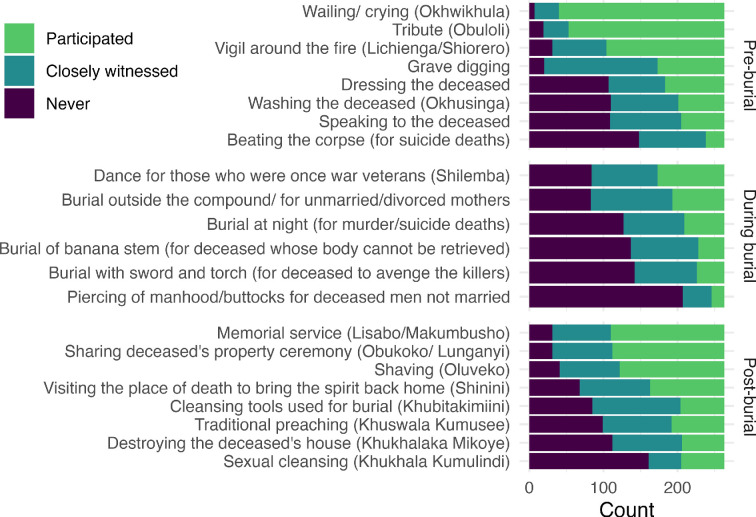
Distribution of responses with regards to participation in pre-burial, burial and post-burial rituals.

Respondents provided information about their participation in 22 mourning rituals, which typically occur prior to burial, during burial or following burial (figure 1). This list was derived from our ethnographic interviews with community members, elders and religious leaders, as well as participant observation in mourning rituals [52]. Respondents were asked whether they had 1 = ‘never participated or witnessed’, 2 = ‘closely witnessed’ or 3 = ‘participated in’ each ritual. Internal consistency for this scale was high (raw alpha = 0.87).

#### (iv) Well-being

Well-being was measured using the modified BBC Subjective Well-Being Scale [62], consisting of three subscales (psychological well-being, physical health and well-being, relationships). While the original test consists of 24 items, we dropped the question ‘Are you happy with access to health services?’ prior to data collection because access to health services is universally limited at the study site. Further, we found that the question ‘Do you feel depressed or anxious?’ was effectively uncorrelated with the rest of the scale (median *r* = 0.041; see electronic supplementary material for further discussion). This is likely because, in the validation of the original scale, Pontin *et al*. [62] did not focus on bereaved individuals; in our case, responses to this question may reflect the immediate emotional impact of recent bereavement rather than overall well-being. We thus opted to remove this item from the main analysis, resulting in a 22-item scale. For each item, respondents provided their answers using a five-point Likert scale: 1 = ‘strongly disagree’, 2 = ‘disagree’, 3 = ‘neutral’, 4 = ‘agree’, 5 = ‘strongly agree’. These responses deviate from those in [62] because the nuance of the original scale (‘not at all’, ‘a little’, ‘moderately’, ‘very much’ and ‘extremely’) could not be accurately captured in Kiswahili. Internal consistency for this scale was high (raw alpha = 0.89), and items were moderately correlated (median *r* = 0.268).

#### (v) Prosociality

Prosociality was measured using the Prosocialness Scale for Adults, a 16-item survey consisting of two subscales (prosocial actions; prosocial feelings) [63,64]. As in the original formulation, respondents provided their answers using a five-point Likert scale: 1 = ‘never/almost never’, 2 = ‘rarely’, 3 = ‘occasionally’, 4 = ‘often’, 5 = ‘always/almost always’. Internal consistency for this scale was high (raw alpha = 0.90), and items were moderately correlated (median *r* = 0.370).

#### (vi) Statistical analyses

**Figure 2.**
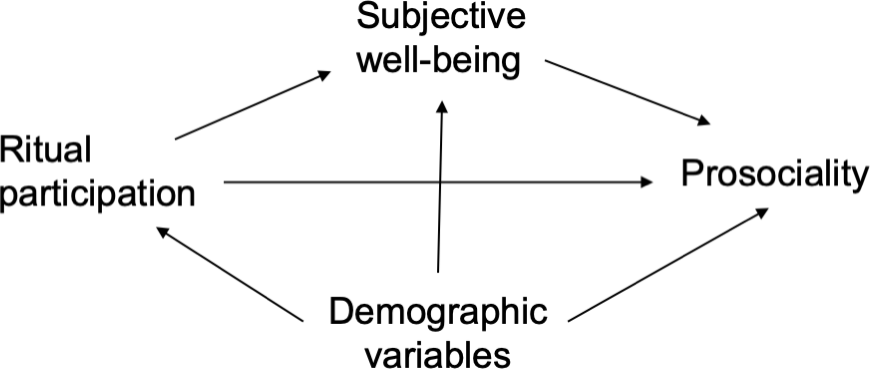
Directed acyclic graph (DAG). This DAG illustrates the proposed causal associations between the main factors investigated in this analysis. Ritual participation is hypothesized to affect prosociality, both directly, and mediated through subjective well-being. We adjust for confounding demographic variables including age, sex, education, occupation and clan.

Analysis was conducted in a Bayesian framework in *Cmdstanr 0.8.1* via *brms* v.2.22.0 in R v.4.4.1 [65]. Our model consisted of two submodels (figure 2). Submodel 1 examined the direct effect of ritual participation on subjective well-being. Submodel 2 examined the indirect effect of ritual participation on prosociality, as mediated by subjective well-being. Scores for each scale were summed and *z*-score standardized, with higher scores reflecting greater levels of ritual participation, well-being and prosociality, respectively. Like many Likert data for relatively short scales, our data were skewed due to respondents responding at or near the boundary values [66]. We thus applied a skew-normal distribution, which is more easily interpretable, and introduces less bias, than transforming the outcome variables [66]. We specified generic weakly informative priors for all variables.

Our ability to infer causal effects is complicated by several compounding issues. First and foremost, our data are observational. While this improves the ecological validity of our results, our findings may be biased by demographic confounds that may covary with ritual participation, subjective well-being and/or prosociality [67,68]. We thus adjusted for the demographic variables outlined above (see also table 1). However, membership to different demographic groups is not random, nor is it balanced across the sample. Further, we cannot assume that confounder effects are independent from each other, nor that their effects are linear. Thus, to improve our inferences, we applied an Inverse Probability of Treatment Weights (IPTW) approach [69], also called ‘independence weights’ in the case of continuous ‘treatments’ such as ritual participation. These methods assign weights that aim to balance the distribution of potential confounders across groups, thus creating a pseudo-population where the predictor of interest (e.g. ritual participation) is statistically independent of confounders like age or clan. Weights were generated in the *WeightIt* R package v.1.3.2 [70] using the energy balancing method [71]. However, in practice, weighting does not offer perfect balance across all covariates, leaving the door open for residual confounding. ‘Double robust’ methods employ both weighting and regression adjustment simultaneously: any remaining imbalance in the confounders can be accounted for with simple parametric terms. Finally, because it can mask the true relationship between variables (i.e. attenuation bias), we incorporated measurement error in our model estimation [68,72].

**Table 1.** Descriptions, and descriptive statistics, for predictor and outcome variables included in this analysis.

variable	description	values	mean (s.d.)
ritual participation^a^	sum of scale responses	1 = never participated/witnessed 2 = closely witnessed 3 = participated	44.138 (8.370)
subjective well-being^a^	sum of Likert responses	1 = strongly disagree 2 = disagree 3 = neutral 4 = agree 5 = strongly agree	83.364 (14.864)
prosociality	sum of Likert responses	1 = never/almost never 2 = rarely 3 = occasionally 4 = often 5 = always/almost always	59.893 (12.03)
age	age category	15−24 years 25−34 years 35−64 years above 64 years	0.356 0.157 0.402 0.084
sex	sex category	male female other	0.414 0.586 0
education	education level	did not attend school primary education secondary education vocational training tertiary education	0.065 0.414 0.387 0.038 0.096
occupation	occupation income level	low middle high	0.808 0.134 0.057
clan	clan territory	Banyala Bashityula Bukusu Batsotso	0.249 0.238 0.268 0.245

^a^ Variables were *z*-score standardized for statistical models.

With these considerations in mind, our model was formulated as follows:


$$\begin{array}{c} {\text{subjective well-being}}_{i}∼\mathrm{SkewNormal}(\xi_{i},\omega ,\alpha )\\ \xi_{i}=\mu_{i}-\omega \delta \sqrt{\frac{2}{\pi }}\\ \mu_{i}=\beta_{0}+\beta_{\text{ritual}}{\text{ritual}}_{i}+\beta \left[{age}_{i}\right]+\beta \left[{sex}_{i}\right]+\beta \left[{education}_{i}\right]\\ \;+\beta \left[{location}_{i}\right]+\beta \left[income_{i}\right]\\ \omega =\frac{\sigma }{\sqrt{1-\frac{2}{\pi }\delta^{2}}}\\ \delta =\frac{\alpha }{\sqrt{1+\alpha^{2}}}\\ \beta_{0},\beta ∼\text{Normal}(0,0.5)\\ \sigma ∼\mathrm{HalfNormal}(0,1)\\ \alpha ∼\mathrm{Normal}(0,2) \end{array}$$

$$\begin{array}{c} {\text{prosociality}}_{i}∼\mathrm{SkewNormal}(\xi_{i},\omega ,\alpha )\\ \xi_{i}=\mu_{i}-\omega \delta \sqrt{\frac{2}{\pi }}\\ \mu_{i}=\beta_{0}+\beta_{\text{ritual}}{\text{ritual}}_{i}+\beta_{SWB}{\text{subjective well-being}}_{i}+\beta \left[age_{i}\right]+\beta \left[{sex}_{i}\right]\\ \;+\beta \left[{education}_{i}\right]+\beta \left[{location}_{i}\right]+\beta \left[{income}_{i}\right]\\ \omega =\frac{\sigma }{\sqrt{1-\frac{2}{\pi }\delta^{2}}}\\ \delta =\frac{\alpha }{\sqrt{1+\alpha^{2}}}\\ \beta_{0},\beta ∼\text{Normal}(0,0.5)\\ \sigma ∼\mathrm{HalfNormal}(0,1)\\ \alpha ∼\mathrm{Normal}(0,2) \end{array}$$

This model was fitted on four chains of 10 000 iterations each. Posterior predictive checks (electronic supplementary material, figure S1) showed that data simulated from the model and the observed data were highly consistent. Further, average residuals were independent of the predictors of interest.

We report 95% CIs throughout. CIs communicate the range of plausible effect sizes. We interpreted the strength of the evidence in favour of our hypotheses using the posterior probability of direction (pd), which quantifies the proportion of the posterior distribution that is consistent with our hypothesis, i.e. the strength of evidence for a directional hypothesis. We use the following thresholds: if pd > 0.95, we consider that ‘strong’ evidence, if pd > 0.8, we consider that ‘moderate’ evidence, if 0.5 < pd < 0.8, we consider that ‘weak’ evidence. We note that these are arbitrary thresholds; we do not use them to guide binary decision-making, but instead to enforce consistent language of interpretation.

### (b) Results

**Figure 3.**
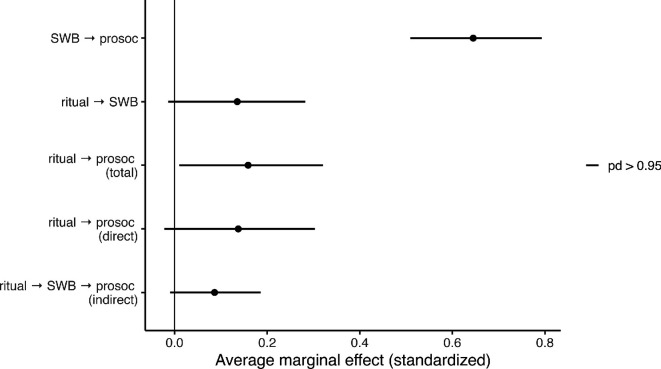
Standardized average marginal effects for hypothesized paths (see figure 2) with points representing posterior means and bars representing 95% CIs. Effects were standardized by dividing both the predictor and response variable by their sample s.d. All estimates derived from double robust Inverse Probability of Treatment Weights (IPTW) with regression adjustment models. SWB=subjective well-being; prosoc=prosociality; ritual=ritual participation.

We found strong support for the hypotheses that ritual participation positively predicts prosociality, and that this relationship is mediated by subjective well-being. Specifically, there was a strong total effect of ritual participation on prosociality. Every 1-unit increase in ritual participation score was associated with an average 0.228 95% CI (0.014, 0.459) increase in prosociality score (pd = 0.982, the total effect). Further, we found strong evidence for the pathway between ritual participation, well-being and prosociality. Every 1-unit increase in ritual participation score was associated with a 0.135 95% CI (−0.014, 0.282) increase in subjective well-being score (pd = 0.964). Each 1-score increase in subjective well-being was associated with a value of 0.522 95% CI (0.412, 0.642) increase in prosociality score, conditional on ritual participation (pd > 0.999). Additional results can be found in figure 3 and electronic supplementary material, table S2.

## 4. Study 2: narrative perspectives on ritual, well-being and cooperation

### (a) Methods

#### (i) Procedure and sample

Focus group discussions and interviews were used to investigate narrative perspectives on ritual, well-being and cooperation. Each focus group consisted of 8−10 participants from homogeneous members of the community to respect the power structures within the Luhya community. Participants were recruited from Kakamega, Vihiga and Bungoma counties. Participants included community elders, bereaved adults and bereaved adolescents, as well as religious leaders. In total, we conducted five focus groups for elders, four for bereaved adults, four for bereaved adolescents and eight in-depth interviews with religious leaders.

The study sample included community elders (67% male, 33% female) aged 65–91 years, bereaved adults (60% male, 40% female) aged 35–75 years and bereaved adolescents (55% male, 45% female) aged 12–21 years. During recruitment, the researchers invited both men and women to participate but observed that slightly fewer women than men signed up. It was also noted during focus group discussions that women appeared passive, with men dominating the conversations. This issue was mitigated by encouraging women to speak when researchers noted male domination. The interview and focus group discussion questions asked participants to describe the mourning rituals practiced by the Luhya community, the relevance of the rituals, their experience after participating in the rituals and how each of the rituals affected community well-being and cooperation. Interviews were conducted in Kiswahili, unless participants requested to be interviewed in Luhya.

Interviews and focus group discussions were recorded using audio recorders, backed up with field notes. Each team consisted of two interviewers, two technical staff to operate the recorders, two note-takers and two observers. This was done to ensure that data were captured by more than one source, thereby assuring the credibility and dependability of the collected data. Audio recordings were transcribed, then translated and back-translated from Kiswahili/Luhya to English. Any inconsistencies between the forward- and back-translations were reviewed, and resolved by discussing and adopting an agreeable translation by consensus.

English-language transcripts were analysed in NVivo v.14. We conducted a thematic analysis following the step-by-step procedure outlined by Braun & Clarke [73]. We identified 14 rituals which we organized into five themes (see table 1 in [52]). For the present paper, we revisited these themes and conducted additional coding for evidence of: (i) well-being, by identifying when participants expressed positive or negative emotions, and (ii) cooperation, by looking for instances where participants talked about coming together, social support and community connections. Following this analysis, we reconvened participants in their original focus groups to confirm that our interpretations accurately reflected their contributions during data collection [74]. We invited participants to give any corrections, clarifications or further reflections, which we subsequently used to refine our interpretations.

### (b) Results

#### (i) Positive impacts of rituals on well-being

Participants reported that social support and warmth were key benefits that could be obtained through performing Luhya traditional mourning rituals. When people gather for vigils around the fire, they support the bereaved family. Similarly, since the family is expected to host many visitors who come to condole with them, they may not be able to accommodate them all. Rituals like the vigil around the fire provide a safe space for people to come together. A Bukusu adult man commented:

*'Every night we spent time at the fire with community members who had come to grieve with us for our father’s death. During the day it felt lonely, but I always felt better when talking with community members seated around the fire at night. The stories we shared kept us laughing all night*.'

A number of respondents attested that participating in rituals gave the bereaved new energy and released pent-up emotions. Some participants believed that the bereaved can faint in the process of burying if they are denied the opportunity to cry; the pain chokes them. A Bukusu elder woman said:

*'Wailing had two reasons, mmh one is releasing that pain or pressure and was a way of informing other people that so and so is gone, so wailing personally I have no problem with it, mmmh even I am one of the preachers who has said to let the people cry because I believe, even in counselling if the person breaks down and cries it’s better because it releases pressure*.'

Besides relief from pent-up emotions, the feeling of having accomplished what was required of them by their culture had a cathartic effect on the mourners. For example, a Bashityula adult woman expressed her joy at the thought of having fulfilled the culture and traditions after witnessing a seated burial. This ritual is done for high-ranking elders who die while still maintaining an esteemed reputation:

*'Like for me I just buried my grandfather sometime back. And it does takes time to bury somebody while seated, it’s not something you can do today then tomorrow no…no…no it does take time, so personally the way we were burying him I saw it brought happiness because we had a lot of people from far, they just wanted to witness if this person was really seated*.'

Cleansing and closure are therapeutic since they allow an individual to continue with their normal life, for instance getting remarried. Members of the community reported that they cannot experience peace if they are faced with unfinished business. Well-being will therefore depend on how the community members are able to close a difficult chapter in their lives and move forward. Respondents attested to the fact that when the rituals are completed, it enhances a sense of acceptance of the loss. When a bereaved participates in a ritual like burying a banana stem (symbolizing a person whose body cannot be traced), they feel better than when they have not buried anything at all. Closure has also been reported after participating in Makumbusho, which is the last remembrance ceremony and final mourning ritual among the Luhya, usually done 1 year after burial. A Banyala elder man said:

*'Let me say this, if somebody is bereaved, he is always confused a little bit, but after carrying out the traditional practices, he comes back to normal the way he was before, he forgets everything because he will have already done makumbusho and he does not think about any other thing, he continues with his normal life*.'

A community works best together if it is reconciled and this is a key aspect that enhances well-being, especially for the living. There are specific mourning rituals done to help reconcile the living and the dead. This leaves the living with a sense of peace. This reconciliation also brings peace to the family. For instance, a Bukusu elder man said:

*'Maybe a parent died while he/she has never eaten in your house, mmmh, you call the brothers to come, you make good food they eat and also you dress them, and assume like your parent is also there. So it happens like that and everything comes back normal? Yeah! So, the dead accepts it, becomes happy and goes well*.'

A Banyala adult woman further explained:

*'When my mother died it was very painful because she had not forgiven me for insulting her… The brothers came and asked me to reconcile with her before burial… I was asked to shake her hand as a sign of peace and her elder brother asked her to forgive me and rest in peace. I felt relieved and my guilt disappeared*.'

#### (ii) Negative impacts of rituals on well-being

Some community members felt that rituals inhibit well-being in various ways. For example, in the Luhya community, a man of child bearing age who dies without a child is buried with a thorn pierced in his manhood, while women are buried outside the compound. Respondents felt that it was not fair to punish a person for issues like childlessness since it was not necessarily the fault of the deceased that they were childless. One Bukusu middle-aged man asked:

*'Why punish a dead woman for childlessness when it was purely a fault of nature? I felt so sad*.'

Respondents with strong religious affiliations to Christianity identified some rituals they felt were associated with demonic connections. They included speaking to the dead, burying someone with a knife and a torch (to facilitate the deceased’s retaliation for their murder), and visiting the place of death to bring the spirit back home (Shinini). This can cause incongruence in the participant. A Batsotso Pastor said:

*'Our church teaching discourages engaging with the spirit of the dead in any way. Once a person has died they need to be totally separated from the living. Communicating with their spirit is divination and totally against the teaching of the bible*.'

There are traditional rituals that intentionally suppress expression of feelings and thus, may not be healthy to the bereaved. In the case of suicide, the children of the deceased are not allowed to attend the funeral nor express their feelings. A Batsotso adult woman said:

*'When children and family members are prevented from crying it hurts more. I have seen people developing problems with breathing and fainting in the funeral when they are trying to suppress their crying*.'

Similarly, for the death of elderly men advanced in age who die before sunset, the bereaved are instructed to delay crying until the evening hours. This may interfere with a family member who may need to express their feelings through crying earlier on. A Bukusu elder woman noted:

*'If it’s an elderly person who died, and has died in the morning, there will be no wailing at that time, they will just cover the body well and wait for his time, presumed as the time when the animals return from the grazing fields, that is, around 3 to 4 p.m. That is now when they will start wailing. Before this time, they just continue with their daily activities and when the time comes for this, they look for their elder son who will be the first one to wail to show respect, and it’s done up to now, even when the body of the elderly is in the mortuary it can’t be brought home before that time*.'

#### (iii) Effect of rituals and cooperation

Rituals are a powerful media for communication among community members, which in turn promote prosociality. Rituals such as wailing, shaving and positioning of graves pass certain messages to the community and beyond. For instance, wailing communicates the occurrence of death. Immediately after wailing begins, community members will gather at the bereaved family’s place, which enhances community cooperation. One Bashitsyula elder man said:

*'…People cry also for other people to know that someone has died*.'

The shaving ritual is done to family members of the bereaved some days after burial. One Batsotso bereaved adult woman reports:

*'If you meet me shaved you will know this one is bereaved. Shaving is a sign that a person is bereaved*.'

Such communication leads community members to suspend their daily activities and join the bereaved family throughout the entire mourning period. Wailing and crying is one way of promoting collective identity, as one Banyala adult woman said:

*'If you hear somebody crying you will go to find out what is happening in that home. This means that everyone is mourning because of the feeling that it is us who are affected. Everyone would mourn, even the neighbours. Even other people from outside the household especially those that this person used to interact with*.'

Rituals such as the vigil around the fire (Shiorero) maintains the community together in collective activities during the mourning period. It gives community members a sense of belonging to the clan. A Batsotso elder man said:

*'Vigil around the fire brings family and community members together to talk/plan how they would bury the dead person*.'

Luhya mourning rituals promote loyalty and attachment to the deceased, maintaining family and community ties even in death. When someone dies, certain cultural practices will be performed to determine the level of loyalty, attachment and detachment towards the dead person. Rituals like naming children after the deceased, memorial ceremonies, returning the body of the deceased to be buried in the ancestral land are some of the rites that not only maintain loyalty to the deceased, but reinforce cooperative behaviour. One Batsotso elder man said:

*'Naming the child happens only in cases where the persons’ deeds/behaviour was good for it is assumed that if you name after a ‘bad’ person, the child will take on the characteristics and behaviours of such a person. Naming a child after a deceased person (whose deeds were good) shows how attached the living were towards the dead*.'

## 5. Discussion

We hypothesized that if participation in mourning rituals boosts cooperation, then mean prosociality scores would be higher for individuals who have participated in traditional Luhya mourning rituals. Furthermore, we hypothesized that if the effect of ritual on prosociality is mediated by well-being, then participation in a traditional Luhya mourning ritual would lead to higher well-being scores which, in turn, would lead to a higher prosociality score. We tested these hypotheses quantitatively. We also explored these dynamics during focus groups and interviews with the bereaved, elders and religious leaders. In what follows, we tie our quantitative and qualitative studies together, and point to avenues for future research.

We found that participating in Luhya mourning rituals strongly increased prosociality. Our qualitative findings further elucidate how rituals boost prosocial behaviour. Specifically, we found that participation in Luhya mourning rituals enhanced communication among community members, promoted collective activities, and solidified loyalty and attachment to the deceased, maintaining family and community ties. Our results echo those from other cultural contexts. For example, Sosis & Ruffle [75] explored how religious rituals in Israeli kibbutzim foster group commitment and trust, revealing that members of religious kibbutzim exhibited greater cooperation than their secular counterparts. This suggests that rituals serve as a mechanism for enhancing social bonds and signalling commitment to group norms. Similarly, the collective nature of mourning rituals can create a shared emotional experience, reinforcing solidarity among participants. Further, the emotional weight of mourning rituals may elicit empathy and altruism, traits that are critical for prosocial behaviour. Xygalatas *et al*. [15,76] found that extreme rituals promoted prosociality through heightened emotional engagement among participants. The shared experience of mourning, characterized by collective grief and remembrance, may similarly enhance empathic responses and encourage supportive behaviours among community members.

We also found a strong pathway between ritual participation, well-being and prosociality. These benefits were supported by our qualitative findings, which showed that participation in Luhya mourning rituals enhances well-being by providing social support, releasing pent-up emotions, creating a cathartic effect on the mourners, providing tranquillity in the community and building reconciliation in the community. This echoes findings from Irving *et al*. [35], who showed that collective rituals can alleviate anxiety and enhance social cohesion. Moreover, according to Saroglou [77], religious and spiritual practices often promote feelings of hope, gratitude and connectedness, potentially contributing to overall life satisfaction. This emotional uplift can enhance well-being, creating a fertile ground for prosocial behaviours to flourish.

It is important to note that the mediating role of well-being in the relationship between ritual participation and prosociality may be influenced by individual differences [78], such as personality traits or prior experiences. Some studies suggest that mourning rituals, while culturally significant, could demean participants and could thus potentially lead to negative emotional states [79]. If participants do not find solace in the ritual, their well-being may not improve, which could hinder the development of prosocial behaviours. In our interviews, we found that some of the rituals were perceived as being punitive, incongruent with the Christian/Muslim beliefs of some community members and were associated with suppression of mourners’ feelings, implying that not all rituals benefit all community members. Thus, our mixed-methods approach presented an opportunity to elevate the voices and experiences of community members who are not in the majority.

Our real-world results offer clear and ecologically valid support to the theoretical relationship between ritual participation and cooperation. As such, our study helps generalize findings from previous field research to an understudied community, the Kenyan Luhya. That the pre-registered hypothesized mediating effect of well-being was strongly supported demonstrates how positive changes in internal states brought on by ritual participation can entrain externally focused prosocial behaviour [13,15,80]. Methodologically, our study shows how ritual cognition and its effects can be meaningfully broken down into simple and measurable psychological phenomena relevant across diverse cultural contexts. Importantly, our findings also suggest that mourning rituals can effectively counter the potential disruption in social cohesiveness associated with the feelings aroused by the death of a community member [37,38]. These findings can thus inform practice in culturally informed clinical psychology. Most local mental health interventions are heavily influenced by Western individualistic approaches [81]. Yet, our participants report that the communal aspects of rituals improve their well-being. Local clinicians could support grieving persons by conducting or facilitating access to rituals. Such an approach would not only improve individual well-being, but, as we’ve shown, can also help grieving individuals connect, cooperate and find support in their broader community. Such interventions—which focus not just on enhancing individual well-being, but also prosocial behaviour—could in theory be protective against complicated grief [82] while helping transition patients more quickly from clinical to community care.

Our study has several limitations. Mediation analyses with observational data make strong assumptions about both the direction of causality and the lack of unmeasured confounders between the exposure (ritual participation), mediator (subjective well-being) and the outcome (prosociality). While we attempted to limit bias by adjusting for demographic variables, there is plausibly residual unmeasured confounding [83]. However, our study offers a degree of socioecological validity that would be difficult or impossible to achieve in an experimental paradigm due to the highly sensitive nature of mourning. Also, all participants were within a year of experiencing the loss of a loved one, which is the timespan during which mourning rituals occur. However, we did not ask participants to report precise timings regarding their loss. While it is possible that more recent losses may evoke stronger emotional responses, the variation in loss timings is likely to be randomly distributed within our sample, making it unlikely that these affect the validity of our statistical results. With a survey covering 22 rituals, we opted to ask participants to respond on a three-point scale to avoid recall biases. That said, the frequency of ritual participation could impact the emotional intensity experienced, something not captured in the present analysis. Our study concentrated on five of the 17 Luhya clans. Future studies will focus on the entire 17 clans to get a wider community perspective on Luhya mourning rituals. Here, we focused on cooperation within the Luhya community, and did not account for in-group and out-group prosocial behaviour [16,21,84]. Future studies will investigate how ritual participation shapes in-/out-group identity. Further, Gelfand *et al*. [25] highlight that while rituals can enhance cooperation, they may also lead to conformity and groupthink, potentially stifling individual expression and reducing creativity. Future work should thus also investigate the trade-offs in individual and group identities during mourning rituals and throughout the mourning period.

## 6. Conclusion

Previous studies have primarily focused on the effect of rituals on prosociality, with linkage to well-being usually overlooked. Our study addressed this gap by examining the association between ritual participation, well-being and cooperation among bereaved Luhya in Kenya. We found strong support for the hypotheses that ritual participation positively predicts prosociality, and that this relationship is mediated by subjective well-being. This pathway was further corroborated by voices of the community through qualitative interviews. Our qualitative data also captured minority voices within the community, who found some of the rituals negatively affected their well-being. Our findings can inform mental health services, where some of the rituals endorsed by the community could be integrated in mainstream psychology as culturally appropriate interventions for grief-related complications.

## Data Availability

Because of the sensitive nature of the data, data will be made available on request from the first author for validation purposes. Analysis code is available on Open Science Framework [85]. Supplementary material is available online [86].
